# Staging liver fibrosis after severe yellow fever with ultrasound elastography in Brazil: A six-month follow-up study

**DOI:** 10.1371/journal.pntd.0009594

**Published:** 2021-07-20

**Authors:** Yuri Costa Sarno Neves, Victor Augusto Camarinha de Castro-Lima, Davi Jorge Fontoura Solla, Vivian Simone de Medeiros Ogata, Fernando Linhares Pereira, Jordana Machado Araujo, Ana Catharina Seixas Nastri, Yeh-Li Ho, Maria Cristina Chammas

**Affiliations:** 1 Radiology Institute, Department of Radiology and Oncology, Hospital das Clinicas HCFMUSP, University of Sao Paulo, São Paulo, Brazil; 2 Department and Division of Infectious and Parasitic Diseases, Hospital das Clinicas HCFMUSP, University of Sao Paulo, São Paulo, Brazil; 3 Department of Neurology, Division of Neurosurgery, Hospital das Clinicas HCFMUSP, University of Sao Paulo, São Paulo, Brazil; NIAID Integrated Research Facility, UNITED STATES

## Abstract

**Background:**

Yellow fever (YF) is a hemorrhagic disease caused by an arbovirus endemic in South America, with recent outbreaks in the last years. Severe cases exhibit fulminant hepatitis, but there are no studies regarding its late-term effects on liver parenchyma. Thus, the aim of this study was to determine the frequency and grade of liver fibrosis in patients who recovered from severe YF and to point out potential predictors of this outcome.

**Methodology/Principal findings:**

We followed-up 18 patients who survived severe YF during a recent outbreak (January-April 2018) in Brazil using ultrasound (US) with shear-wave elastography (SWE) at 6 months after symptoms onset. No patient had previous history of liver disease.

Median liver stiffness (LS) was 5.3 (4.6–6.4) kPa. 2 (11.1%) patients were classified as Metavir F2, 1 (8.3%) as F3 and 1 (8.3%) as F4; these two last patients had features of cardiogenic liver congestion on Doppler analysis. Age and cardiac failure were associated with increased LS (*p = 0*.*036* and *p = 0*.*024*, respectively). SAPS-3 at ICU admission showed a tendency of association with significant fibrosis (≥ F2; *p = 0*.*053*). 7 patients used sofosbuvir in a research protocol, of which none showed liver fibrosis (*p = 0*.*119*).

**Conclusions/Significance:**

We found a low frequency of liver fibrosis in severe YF survivors. US with SWE may have a role in the follow up of patients of age and / or with comorbidities after hospital discharge in severe YF, a rare but reemergent disease.

## Author summary

Yellow fever (YF) is a viral disease transmitted by mosquitoes and represents an important health problem in countries of South America and Africa, with recent outbreaks in the past few years. Severe cases lead to fulminant hepatitis and death; it has also been reported that surviving patients tend to recover / “regenerate” the liver after the acute phase of the disease. However, there are no prior investigations on this matter. Thus, we followed up a group of previously healthy patients who had severe YF 6 months after the initial symptoms, using a non-invasive ultrasound technique of estimating the liver fibrosis (scar tissue) grade, called elastography. In our findings, we report a low frequency of liver fibrosis. Thus, we concluded that patients who had severe YF are not likely to have late-term liver fibrosis or cirrhosis. Nevertheless, there are some specific individuals (older or with chronic diseases) that might need further evaluation.

## Introduction

Yellow fever (YF) is a mosquito-borne hemorrhagic disease caused by an arbovirus of genus Flavivirus that occurs in tropical regions of South America and Africa [1]. In Brazil, urban transmission cycle was interrupted in 1940’s due to massive vaccination campaign and vector control programs [2]. However, the enzoosis remains present and human infection continues to occur in a sylvatic cycle [3]. In the beginning of 2018, a YF outbreak occurred on the outskirts of Sao Paulo; 538 cases were confirmed between January and December 2018, with a lethality rate of 34.6%[4]. The pathophysiology of the disease involves viral tropism and acute direct damage to vital organs such as liver and kidneys[5–7], in severe cases leading to fulminant hepatitis and acute renal failure (often requiring replacement therapies)[8–11], with high in-hospital case fatality rates (of up to 67%, according to recent studies[12–14]).

It is not clear whether liver fibrosis arises after initial insult in severe form surviving patients. Some reports showed that patients recover completely from other acute hepatitis, with no signs or symptoms of chronic hepatitis or cirrhosis[15,16]. However, specific longitudinal data in YF patients after recovery is lacking. Due to the rarity of the disease and the absence of specific technologies in prior outbreaks, few patients were adequately followed up in order to clarify this matter. Additionally, a recent series demonstrated a late-onset relapsing hepatitis, a newly discovered entity, in patients who recovered from the acute phase of the disease[17].

In this context, non-invasive assessment of liver fibrosis with ultrasound (US) using new shear-wave elastography (SWE) techniques can be an important and accessible tool to investigate liver parenchyma status after acute hepatitis[18]. US-based elastography techniques are low-cost and radiation-free imaging methods[19], with performances comparable to those of biopsy[20]. It is also worth noting that liver biopsy with histological examination, although historically considered the reference standard for staging fibrosis, has considerable interobserver variability[21] and the disadvantage of being an invasive procedure, with intrinsic risks, and thus not suitable for longitudinal monitoring. Hence, the aim of this study was to determine whether patients who recovered from severe yellow fever developed liver fibrosis based on SWE evaluation and to point out potential predictors (clinical and laboratory) of cirrhosis during the follow up time.

## Methods

### Ethics statement

All enrolled patients signed an Informed Consent Form. Research protocol was approved by the Ethics Committee of Hospital das Clínicas da Faculdade de Medicina da Universidade da Universidade de São Paulo–HCFMUSP—CAPPesq (n^o^ 2.833.376). We followed the STROBE Statement checklist for observational studies [22].

### Study design

This is a cohort study of patients who were admitted to an infectious diseases intensive care unit (ICU) of a teaching tertiary-care hospital in São Paulo (Hospital das Clínicas, University of São Paulo) with the diagnosis of severe YF, confirmed by serum polymerase chain reaction (PCR). Criteria for severe YF and to be admitted to the ICU were pre-specified in a prior study[23]. Individuals discharged between January and April 2018 were contacted by phone, invited to participate in the study and to attend to the Hospital’s Ultrasonography Department to perform a non-enhanced abdominal US with SWE. Scans were carried at 6 months after symptoms onset. There were no exclusion criteria.

SWE evaluations were performed by a board-certified radiologist (5 years of training in US) on an RS80A US system (Samsung Medison Co., Ltd.) equipped with S-SWE (Samsung shear-wave elastography, a type of point shear wave technique) using a convex probe (CA1-7A). All patients fasted for at least 4 hours prior to the examination. Liver stiffness (LS) measurements, expressed in kilopascals (kPa), were done using an intercostal approach, with the patient placed in a supine position with the right arm maximally abducted above the head and performing expiratory breath-holds. Regions of interest (ROIs) were placed in the right lobe of the liver at a depth of approximately 2–4 cm from its capsule, avoiding large vessels. At least 5 valid measurements were obtained, and the median LS value was taken as a representative value if, after consecutive measurements, an interquartile range (IQR)/median ratio ≤ 30% was achieved. S-SWE technique also automatically calculated a reliability measurement index (RMI), which demonstrated the reliability of each measurement. The RMI ranged from 0 to 1.0, with an RMI ≥ 0.4 being considered acceptable, according to the manufacturer’s instruction. We used the cutoffs for staging fibrosis (correlating to Metavir scoring system) of 7 kPa for ≥ F3 and 9,7 kPa for F4 [24–26]. Scanning protocol also included grayscale (B-mode), color and spectral Doppler modes. Data acquired were archived in PHILIPS iSite Picture Archiving and Communication Systems (PACS) version 4.1.110.0. Each patient had at least 30 images acquired; there were no videos available.

Clinical, demographic and anthropometric data were collected during a short interview and also acquired from medical records (including the Simplified Acute Physiology Score 3—SAPS-3). Laboratory tests were performed in the same day of US elastography examination (and also retrieved from medical records from previous ICU admission) and included liver enzymes, bilirubin levels, serum creatinine and urea, platelets, prothrombin time and international normalized ratio (INR).

### Statistical analyses

For descriptive purposes, categorical variables were presented through relative and absolute frequencies and compared by means of the chi-squared or Fisher exact test, as appropriate. Continuous variables distributions were assessed for normality by skewness and kurtosis and graphical methods. Those with normal distribution were presented as mean and standard deviations and compared by the independent samples Student T test. Otherwise, they were presented as medians and quartiles and compared by the Mann-Whitney non-parametric test. In order to address the association between liver stiffness and demographic and laboratory variables, correlation analysis was performed through the Spearman method, since US SWE parameters were non-normally distributed.

All tests were two-tailed and final p values under 0.05 were considered statistically significant. All analyses were conducted with the SPSS software (IBM Corp. SPSS Statistics for Windows, version 24.0. Armonk, NY).

## Results

**Fig 1:**
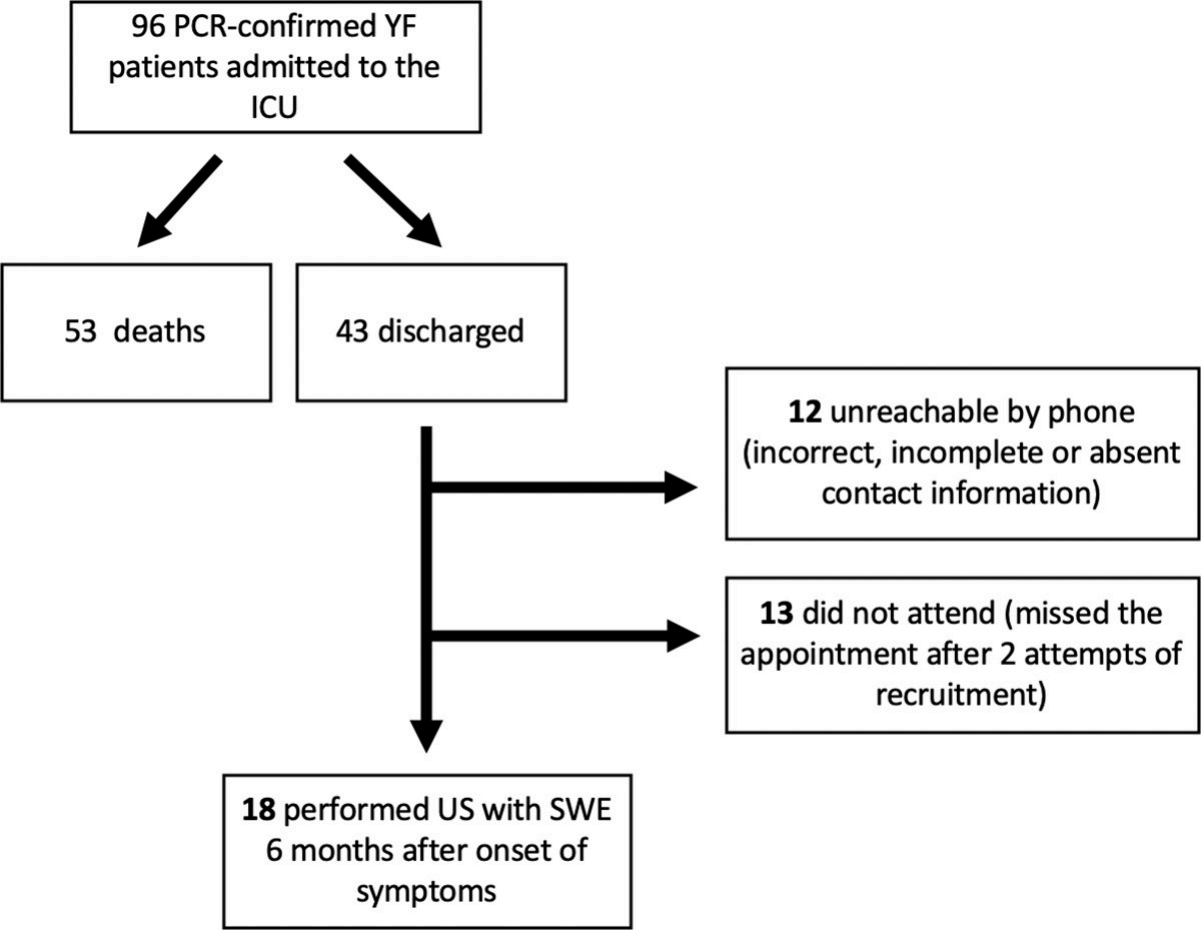
Flow diagram of patient enrolment. Of the 43 patients who survived severe yellow fever and were discharged, we managed to evaluate 18 patients with ultrasound elastography after 6 months of symptoms onset. Reasons for the losses of follow-up are specified on the right. PCR = Polymerase chain reaction. YF = Yellow fever. ICU = Intensive care unit. US = Ultrasound. SWE = Shear-wave elastography.

Eighteen individuals were included in the study and performed an evaluation with elastography at a median time of 185 (180–191) days after onset of symptoms. **Fig 1** shows patient enrolment. Losses were due to incorrect or incomplete contact information or no attendance after 2 attempts of recruitment. Fifteen (83.3%) patients were male; median age was 47 (41–57) years and median body mass index was 23.6 (21.6–24.8) kg/m^2^. No patient had prior history of liver disease or was submitted to liver transplantation. Serologies for hepatitis A, B and C were negative for all patients. Two patients (11.1%) had been vaccinated for YF less than 10 days before admission. None was considered to have vaccinal disease, because only wild virus was detected with PCR. Median length of hospital stay was 11 days (8–23) and median SAPS-3 at admission was 49 (45–60). At admission to the UCI, laboratory tests revealed high serum levels of aspartate aminotransferase, alanine aminotransferase and total bilirubin (medians of 3479 U/L, 2531 U/L and 4.37 mg/dL, respectively), median serum creatinine of 1.43 mg/dL (1.06–4.59 mg/dL) and median serum urea concentration of 62 mg/dL (26–124 mg/dL). Median INR was 1.23 (1.10–1.40), and median platelet count was 55 units/mm^3^ (40.000–72.000 units/mm^3^). Lab results at 6 months after symptoms onset were within normal ranges. Seven (38.9%) patients used sofosbuvir for 10 days in a research protocol during the hospitalization [17,27]. **Table 1** shows clinical, demographic, laboratory and elastography data.

**Table 1:** Clinical, demographic, laboratory and elastography data of 18 patients who had severe yellow fever and were followed up to 6 months after onset of symptoms (descriptive statistics), Brazil, 2018*.

Variable	N = 18
**Age** (years) (mean ± SD)	45.9 ± 14.8
Median and quartiles	47 (41–57)
Range	19–69
**Male gender**	15 (83.3)
**Body mass index** (median and quartiles)	23.6 (21.6–24.8)
**Hypertension**	4 (22.2)
**Diabetes**	3 (16.7)
**Cardiac failure**	2 (11.1)
**Alcohol use (current)**	9 (50.0)
**SAPS-3 at admission** (median and quartiles)	49 (45–60)
**Length of hospital stay** (days) (median and quartiles)	11 (8–23)
**Sofosbuvir use**	7 (38.9)
***Laboratory results at admission***	
**AST** (U/L) (Median and quartiles)	3479 (2260–4124)
**ALT** (U/L) (Median and quartiles)	2531 (1790–2815)
**Total bilirubin** (mg/dL) (median and quartiles)	4.37 (1.99–8.06)
**Alkaline phosphatase** (U/L) (median and quartiles)	106 (100–132)
**GGT** (U/L) (median and quartiles)	279 (211–533)
**Urea** (mg/dL) (median and quartiles)	62 (26–124)
**Creatinine** (mg/dL) (median and quartiles)	1.43 (1.06–4.59)
**INR** (median and quartiles)	1.23 (1.10–1.40)
**Platelets** (1000 units/mm^3^) (median and quartiles)	55 (40–72)
***Laboratory results at 6 months after onset of symptoms***	
**AST** (U/L) (median and quartiles)	24 (21–26)
**ALT** (U/L) (median and quartiles)	27 (19–37)
**Total bilirubin** (mg/dL) (median and quartiles)	0.45 (0.30–0.61)
**Alkaline phosphatase** (U/L) (median and quartiles)	71 (61–77)
**GGT** (U/L) (median and quartiles)	31 (23–51)
**Urea** (mg/dL) (median and quartiles)	32 (26–37)
**Creatinine** (mg/dL) (median and quartiles)	0.87 (0.83–1.11)
**INR** (median and quartiles)	0.99 (0.96–1.03)
**Platelets** (1000 units/mm^3^) (median and quartiles)	207 (175–246)
**Time since onset of symptoms and elastography** (days) (median and quartiles)	185 (180–191)
**Shear wave velocity** (m/s) (median and quartiles)	1.33 (1.24–1.46)
**Liver stiffness** (kPa) (median and quartiles)	5.3 (4.6–6.4)
**Liver fibrosis categories (METAVIR)**	
F0/F1	14 (77.8)
F2	2 (11.1)
F3	1 (5.6)
F4	1 (5.6)

*Data presented as no. (%), except if specified otherwise. SD, Standard deviation. SAPS-3, Simplified Acute Physiology Score 3. AST, aspartate aminotransferase. ALT, alanine aminotransferase. GGT, gamma-glutamyl transferase. INR, international normalized ratio.

**Fig 2:**
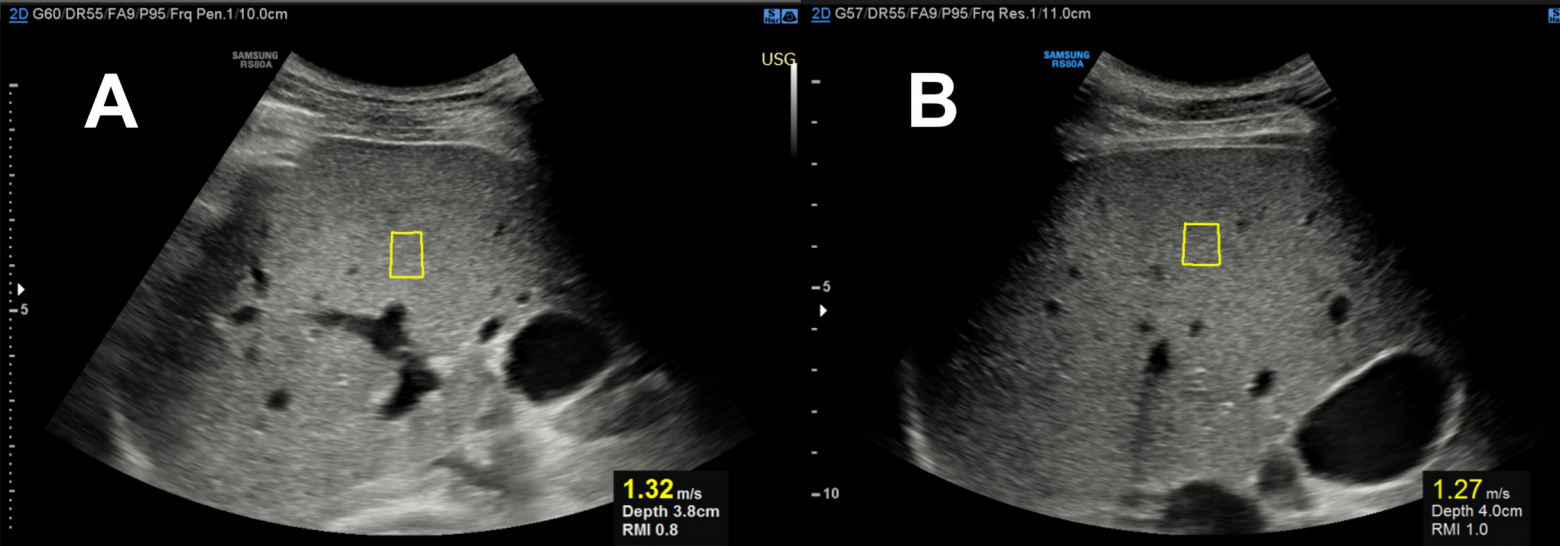
Right upper quadrant ultrasound of two patients using point shear-wave elastography technique. (A) A 48-year-old male patient, and (B), a 38-year-old male patient, both asymptomatic, 6 months after onset of severe yellow fever. Images depict placement of regions of interest (ROIs) on the right lobe of the liver at expiratory breath-hold. Shear-wave velocities (in m/s) were generated, with corresponding liver stiffness values after multiple measurements of 5.3 kPa and 4.6 kPa, respectively. Both subjects were then classified as F0/F1 on the Metavir scoring system for staging of fibrosis. RMI = Reliability measurement index.

Regarding US findings with elastography evaluation (SWE) (**Fig 2**), median shear-wave velocity and LS were 1.33 m/s (1.24–1.46) and 5.3 (4.6–6.4) kPa, respectively. Two (11.1%) patients had mild fibrosis (F2), one (8.3%) had moderate fibrosis (F3) and one (8.3%) had cirrhosis (F4); these two last patients had signs of congestive heart failure, with features of cardiogenic liver congestion on Doppler analysis. All remaining patients had Doppler evaluation of superior abdomen (portal vein, hepatic veins and hepatic artery) within normal ranges. No patient showed morphologic changes of cirrhosis on grayscale mode (**Table 1**).

**Fig 3:**
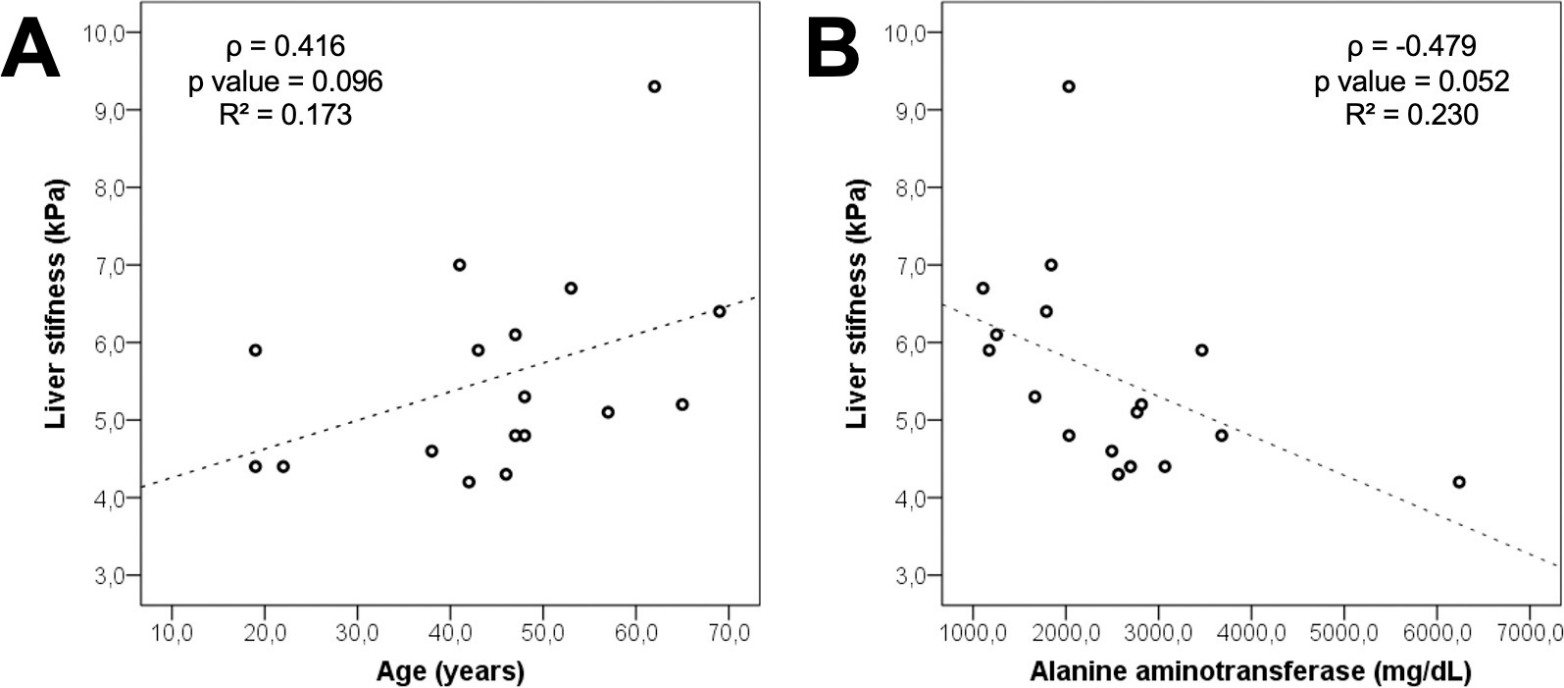
Correlations between liver stiffness and clinical and laboratorial variables. Age (A) and alanine aminotransferase (B) and its correlations with liver stiffness, using the Spearman method (levels of significance of p = 0.096 and p = 0.052, respectively). kPa = Kilopascals.

**Table 2** compares clinical and demographic variables and laboratory results at admission with LS values obtained 6 months after onset of symptoms. Heart failure was associated with increased LS (*p = 0*.*024*); systemic arterial hypertension and diabetes showed a tendency towards this association *(p = 0*.*063* and *p = 0*.*066*, respectively). After exclusion of the outlier patient (LS = 22,4 kPa, with cardiogenic liver congestion), there was a tendency towards statistical significance in the correlations between LS and age (ρ = 0.416, *p = 0*.*096*) and between LS and alanine aminotransferase at admission (ρ = -0.479, *p = 0*.*052*) (**Fig 3**). We found no significant association between LS and gender, BMI, alcohol consumption, hospitalization time or other laboratorial results.

**Table 2:** Differences in fibrosis estimations of 18 patients who had severe yellow fever 6 months after onset of symptoms according to clinical and demographic characteristics, Brazil, 2018.

Variable		Liver stiffness (kPa) (median and quartiles)	p value
**Gender**	Female	6.40 (5.90–22.40)	0.075
	Male	5.10 (4.40–9.30)	
**Hypertension**	Yes	7.60 (5.55–22.40)	0.063
	No	4.95 (4.40–7.00)	
**Diabetes**	Yes	9.30 (5.20–22.40)	0.066
	No	5.10 (4.40–7.00)	
**Cardiac failure**	Yes	15.85 (9.30–22.40)	**0.024**†
	No	5.15 (4.50–7.00)	
**Alcohol use (current)**	Yes	5.90 (5.20–9.30)	0.102
	No	4.80 (4.40–22.40)	
**Sofosbuvir use**	Yes	4.60 (4.40–6.40)	0.070
	No	5.90 (4.80–22.40)	

†Statistical significance.

Considering the cutoff for significant fibrosis (≥ F2), differences in clinical and laboratory data during hospitalization are shown in **Table 3.** Cardiac failure was associated with a Metavir score ≥ F2 (50% versus 0; *p = 0*.039). There was a tendency towards an association between higher SAPS-3 values at hospital admission and significant fibrosis at 6 months after onset of symptoms (medians of 71 versus 47; *p = 0*.*053*). There were no statistically significant differences in the frequencies or median values of other variables between these two groups of participants.

**Table 3:** Clinical and laboratory data at hospitalization according to liver fibrosis staging (with or without significant fibrosis) of 18 patients at 6 months after onset of symptoms of severe yellow fever, Brazil, 2018.

Variable	Fibrosis stage		
	F0/1 (n = 14)	≥ F2 (n = 4)	p value
**Age** (years) (median and quartiles)	46.5 (38.0–48.0)	57 (47–61.5)	0.242
**Male gender**	12 (85.7)	3 (75.0)	1.000
**Body mass index** (median and quartiles)	22.7 (21.3–24.3)	27.8 (23.2–31.8)	0.151
**Hypertension**	2 (14.3)	2 (50.0)	0.197
**Diabetes**	1 (7.1)	2 (50.0)	0.108
**Cardiac failure**	0	2 (50.0)	**0.039**†
**Alcohol use (current)**	6 (42.9)	3 (75.0)	0.576
**SAPS-3 at admission** (median and quartiles)	47 (42–60)	71 (54–73)	*0*.*053*
**Length of hospital stay** (days) (median and quartiles)	10 (8–25)	18 (12–21)	0.709
**Sofosbuvir use**	7 (50.0)	0	0.119
**AST** (U/L) (median and quartiles)	3238 (2260–4124)	3518 (2327–4102)	0.915
**ALT** (U/L) (median and quartiles)	2631 (1790–3067)	1937 (1475–2410)	0.243
**Total bilirubin** (mg/dL) (median and quartiles)	3.2 (2.0–8.1)	6.1 (3.3–14.9)	0.524
**Alkaline phosphatase** (U/L) (median and quartiles)	105 (100–129)	140 (89–184)	0.524
**GGT** (U/L) (median and quartiles)	261 (211–533)	371 (174–566)	0.791
**INR** (median and quartiles)	1.2 (1.1–1.4)	1.3 (1.2–1.7)	0.396
**Fibrinogen** (mg/dL)	133 (121–146)	148 (124–215)	0.594
**Factor V** (%)	92 (65–133)	118 (67–132)	0.873

*Data presented as no. (%), except if specified otherwise.

†Statistical significance. SAPS-3, Simplified Acute Physiology Score 3. AST, aspartate aminotransferase. ALT, alanine aminotransferase. GGT, gamma-glutamyl transferase. INR, international normalized ratio.

No patient who received sofosbuvir showed liver fibrosis at 6 months after onset of symptoms, compared with 4 (36.4%) individuals who had fibrosis (≥ F2) in the group that did not receive the drug. This difference did not reach statistical significance.

## Discussion

This is a unprecedent investigation on this rare but reemergent and severe disease. Our study managed to determine the frequency of liver fibrosis in severe YF surviving patients, utilizing a modern US SWE technique, with good reproducibility and correlation to liver biopsy[18]. High grades of LS were found in a small percentage of patients, similar to the prevalence in the general population[28–30], indicating that the liver recovers at least in great portion after the initial viral insult (analogous to what was suggested in prior reports for other viral hepatitis[15,16]). Thus, US with SWE showed to be a valid an noninvasive method to evaluate these patients, without the disadvantages of conventional biopsy[31].

Nevertheless, some clinical characteristic seemed to be associated with increased LS. Older age was predictably correlated to higher LS values, as expected; chronic diseases such as diabetes and hypertension also demonstrated a tendency towards this association. We found a tendency to a negative correlation between alanine aminotransferase at admission and LS; it is more likely that this association be spurious than a real and protective factor. Higher SAPS-3 showed a tendency of an association with significant fibrosis and could potentially represent a predictor of long-term fibrosis. Cardiac failure, a condition that determines hepatic congestion, was present in two of 18 patients at 6 months after onset of symptoms, both of which had increased LS; however, this result was most likely a “false-positive”, since edema of liver tissue leads to increased stiffness not related to fibrous tissue[32]. For a similar reason, acquiring a “baseline” SWE at hospitalization would not be valid (nor feasible) due to inflammation and edema of the hepatic parenchyma during the acute phase, also associated to increased LS [16,33,34]. No other clinical or laboratory variable showed to be a predictor of fibrosis.

Sofosbuvir administration during the hospitalization period, as a part of a randomized clinical trial for treatment of acute YF occurring concomitantly in our institution[27], might have altered the proportions of fibrosis during the follow up period. Corroborating with this is the fact that no patient that used sofosbuvir was categorized as having significant fibrosis at the time of completion of 6 months after onset of symptoms (although that difference did not reach statistical significance). There was also a report of high incidence of late-onset relapsing hepatitis in these patients (that is, a new elevation in aminotransferase levels within 6 months after an improvement in or normalization of liver function, occurring in up to 45%, according to a recent study[17]), but this have not seemed to alter our results.

No patient in our sample was adequately immunized for YF or had vaccinal disease. It is important to note that, by the time of this outbreak, the city of São Paulo and its outskirts were not yet included in the areas of universal (i.e. permanent) YF vaccination, presenting a low vaccination coverage [35,36].

Some limitations of this work must be acknowledged. The small sample size, inherent to the epidemiology of the disease, must be taken into account. Although not sufficient to ensure adequate power to the statistical analysis and to confirm the hypothesis, it retains great relevance and represents a unique window of opportunity in the context of a rare disease, never yet studied in this scale. The observational nature of the investigation brings a series of usual bias, and thus we tried to avoid them by following strict rules and protocols. There was no control group whatsoever; we then needed to compare our findings to populational statistics, not always comparable (e.g., with the same characteristics) to our group of individuals. The lack of a baseline evaluation, in turn, can be justified by the aforementioned argument–it would not be possible to evaluate patients at the acute phase of yellow fever with SWE since acute inflammation changes (increases) LS. It is also of note that no patient had prior history of liver disease and hepatitis viruses were ruled out. There were many losses of follow up due to logistic problems, lack of contact information or no attendances, and this might have affected the results. Furthermore, survival bias is likely to have occurred, since patients that died at hospital could have had a higher probability of developing liver fibrosis if fatal outcome have not happened. Also, there are still no trustworthy cutoffs for fibrosis using acoustic radiation force impulse (SWE techniques) by different fabricators and to different diseases[18,37,38], hence we preferably used the absolute values of LS (continuous variable) to express our results.

In conclusion, we believe our work increases the knowledge of the natural history of this rare disease. US can be a tool to evaluate the liver in patients during the acute phase of severe YF, as it was demonstrated in our previous study [23], but can also be of value and have a role in the follow up of patients of age and / or with comorbidities after hospital discharge. Increased SAPS-3 might be associated with liver fibrosis after survival, but this result needs to be confirmed by further studies.

## Supporting information

S1 STROBE checklist (DOCX)

## Abbreviations

ICU: intensive care unit
kPa: kilopascals
LS: liver stiffness
SWE: shear-wave elastography
US: ultrasound
YF: yellow fever
