# Supplementary material for: Staging liver fibrosis after severe yellow fever with ultrasound elastography in Brazil: A six-month follow-up study
Source: PLoS Negl Trop Dis. 2021 Jul 20;15(7):e0009594. doi: 10.1371/journal.pntd.0009594 (PMC8323872; doi:10.1371/journal.pntd.0009594)
Supplement: Attachment — Submitted filename: Response Letter to the Editor and Reviewers.docx [file pntd.0009594.s002.docx]

Manuscript Number: **PNTD-D-21-00158** – *Review Response*.

Dear Michael R Holbrook and Elizabeth Carlton, members of the editorial board and independent reviewers:

We thank you very much for the consideration and effort put in revising our manuscript. We also fully understand the extended period needed to complete this task of yours, given the current global circumstances.

We are very happy for your interest in our work and appreciated the important reviews. Based on its recommendations and instructions, we sought to modify our Paper accordingly. The feedback from you has helped us to significantly improve our manuscript.

This is our point-by-point rebuttal. We put maximum effort into answering all the suggestions and recommendations of the reviewers.

Best regards,

Yuri Neves

Corresponding Author

Hospital das Clínicas Universidade de São Paulo

***Regarding the preliminary topics mentioned in the Decision Letter (Editorial board):***

Comment 1. Important comments regarding vaccination status of the patients included in the study.

**Response to Comment 1. Indeed, important observation. We addressed this topic in the Response to Reviewer 2, comment 2.**

Comment 2. Need to acknowledge the limitations of the small sample size.

**Response to Comment 2. Agreed. We added a comment in the Discussion section, acknowledging in more detail this limitation and its meaning to the study interpretation.**

Comment 3. Analysis of the association between LS and lipase should address the influence of the outlier value.

**Response to Comment 3. Thanks for the remarks.**

**The subject with the highest LS (22,4 kPa) had cardiac liver congestion, which increases parenchymal stiffness.**

**The value previously registered for the subject with the highest lipase was mistaken, and thus we corrected this value.**

**We then performed a new analysis correcting and removing the outliers.**

Comment 4. Statistical methods sections notes the use of GLM for repeated measures and examination of interactions but it appears each person had only one SWE evaluation and no discussion of interactions are presented in the results.

**Response to Comment 4. Thanks for pointing out this mistake. Actually, we had performed a previous and preliminary analysis considering elastography evaluations acquired in non standardized points of time in a portion of our sample (prior to 6 months, in some patients).**

**However, we chose not to include this analysis, since it was adding unnecessary complexity without an increase in useful information. Thus, in the final version, for the sake of manuscript clarity, this data was not shown (but we forgot to remove its description in the Methods section).**

**Hence, we removed the aforementioned paragraph from the Revised Manuscript.**

Comment 5. Clarify the statistical analyses methods and ensure they align with the analyses presented in the results (it can be useful to start the statistical analysis methods by reminding the reader the question(s) you are trying to address through statistical analysis).

**Response to Comment 5. Excellent suggestion. We modified the Methods section so it can address this clarification.**

***Response to Reviewer 1:***

Thanks for your comments. We sincerely appreciated the considerations on the major strengths and weaknesses of our work. Indeed, we recognize this is a unprecedent investigation with interesting results, despite its small sample size (inherent to the epidemiology of this uncommon disease). **We added a comment in the Discussion section, acknowledging in more detail this limitation and its meaning to the study interpretation.**

Much effort was put in the statistical section to derive adequate conclusions of our data. We hope this paper brings more clarity to the physiopathology of this tropical and neglected disease.

***Response to Reviewer 2:***

We again appreciate the detailed review, with important considerations. This is our point-by-point rebuttal.

Rev. 2, comment 1. Interesting work about a relatively rare disease. However, due to the small sample the study can only generate hypothesis.

**Response to Rev. 2, comment 1. Important observation. We added a comment in the Discussion section, acknowledging in more detail this limitation and its meaning to the study interpretation.**

Rev. 2, comment 2. Considering that yellow fever is a vaccine-preventable disease and São Paulo is an area with a yellow fever vaccine recommendation, would be interesting to know about the vaccine status of the study subjects.

**Response to Rev. 2, comment 2. Good recommendation, thanks. We added the vaccination status of all study subjects in Results section and discussed it further in Discussion section.**

**It is also important to note that, by the time of the 2018 outbreak, the state of São Paulo had not yet been assigned a universal vaccination recommendation for YF (as shown in the 2017 document from our Ministry of Health – new reference 36). São Paulo was still considered an area of temporary vaccination (hence not in the permanent vaccination schedule).**

Rev. 2, comment 3. Please note that some terms need to be revised:

In the line 137, please review the term mortality (it’s probably lethality).

In the line 141, please review the term mortality rate (maybe “case fatality rate”).

Line 257 – lab results WERE.

**Response to Rev. 2, comment 3. Thanks for the attention to detail. We revised the terms in the above mentioned lines.**

Rev. 2, comment 4. In the line, 394, please review the reference. “Prof. Alexandre Vranjac” is the name of the Centro de Vigilância Epidemiológica, not the author of the reference.

**Response to Rev. 2, comment 4. Thanks. We corrected this reference.**
