# Supplementary material for: Staging liver fibrosis after severe yellow fever with ultrasound elastography in Brazil: A six-month follow-up study
Source: PLoS Negl Trop Dis. 2021 Jul 20;15(7):e0009594. doi: 10.1371/journal.pntd.0009594 (PMC8323872; doi:10.1371/journal.pntd.0009594)
Supplement: Peer review history

# Peer review: Decision Letter 0

**DOI:** 10.1371/journal.pntd.0009594.r001

**Authors:** Elizabeth J Carlton, Michael R Holbrook

1 Jun 2021

Dear Dr. Neves,

Thank you very much for submitting your manuscript "Staging liver fibrosis after severe yellow fever with ultrasound elastography in Brazil: A six-month follow-up study" for consideration at PLOS Neglected Tropical Diseases. As with all papers reviewed by the journal, your manuscript was reviewed by members of the editorial board and by several independent reviewers. The reviewers appreciated the attention to an important topic. Based on the reviews, we are likely to accept this manuscript for publication, providing that you modify the manuscript according to the review recommendations. 

The reviewers raised important comments regarding vaccination status of the patients included in the study and also the need to acknowledge the limitations of the small sample size. These should be addressed in the revision. In addition, we request two clarifications to the statistical analyses. The analysis of the association between LS and lipase should address the influence of the outlier value. The statistical methods sections notes the use of GLM for repeated measures and examination of interactions but it appears each person had only one SWE evaluation and no discussion of interactions are presented in the results. Please clarify the statistical analyses methods and ensure they align with the analyses presented in the results (it can be useful to start the statistical analysis methods by reminding the reader the question(s) you are trying to address through statistical analysis).

We would like to apologize for the extended period it took to complete the review of your manuscript. Given the current international health crisis, it is taking some time to identify and complete reviews.

Please prepare and submit your revised manuscript within 30 days. If you anticipate any delay, please let us know the expected resubmission date by replying to this email. 

When you are ready to resubmit, please upload the following:

[1] A letter containing a detailed list of your responses to all review comments, and a description of the changes you have made in the manuscript. 

Please note while forming your response, if your article is accepted, you may have the opportunity to make the peer review history publicly available. The record will include editor decision letters (with reviews) and your responses to reviewer comments. If eligible, we will contact you to opt in or out

[2] Two versions of the revised manuscript: one with either highlights or tracked changes denoting where the text has been changed; the other a clean version (uploaded as the manuscript file).

Important additional instructions are given below your reviewer comments. 

Thank you again for your submission to our journal. We hope that our editorial process has been constructive so far, and we welcome your feedback at any time. Please don't hesitate to contact us if you have any questions or comments.

Sincerely,

Michael R Holbrook, PhD

Associate Editor

PLOS Neglected Tropical Diseases

Elizabeth Carlton

Deputy Editor

PLOS Neglected Tropical Diseases

***********************

We would like to apologize for the extended period it took to complete the review of your manuscript. Given the current international health crisis, it is taking some time to identify and complete reviews.

Reviewer's Responses to Questions

**Key Review Criteria Required for Acceptance?**

As you describe the new analyses required for acceptance, please consider the following:

**Methods**

-Are the objectives of the study clearly articulated with a clear testable hypothesis stated?

-Is the study design appropriate to address the stated objectives?

-Is the population clearly described and appropriate for the hypothesis being tested?

-Is the sample size sufficient to ensure adequate power to address the hypothesis being tested?

-Were correct statistical analysis used to support conclusions?

-Are there concerns about ethical or regulatory requirements being met?

Reviewer #1: The manuscript present a unique opportunity to evaluate the convalescent phase of a uncommon disease such a Yellow fever. As this disease presents sporadically, the chance to take direct measures of clinical parameters presents in a constrict time window that must be used, in order to better understand how the pathophysiology develops and have under-notice effects on this survivors.

With a reasonable number of UCI survivors of a disease with such a high reported mortality rate, the authors deliver a compelling argument substantiated with rigorous measurements and statistics.

All patients signed the informed consent and the projects was approved by the corresponding ethics committee.

The conclusions are interesting and solid.

I do recommend the present work for publication without hesitation.

Reviewer #2: The historical cohort design is suitable for assessing risk factors and the evolution / outcome of yellow fever. The sample size is probably not sufficient to ensure adequate power to the statistics analysis but the small sample is inherent to the epidemiology of the disease. No ethical concerns were identified.

--------------------

**Results**

-Does the analysis presented match the analysis plan?

-Are the results clearly and completely presented?

-Are the figures (Tables, Images) of sufficient quality for clarity?

Reviewer #1: My criteria was included in the Instructions section.

Reviewer #2: The results were presented properly.

--------------------

**Conclusions**

-Are the conclusions supported by the data presented?

-Are the limitations of analysis clearly described?

-Do the authors discuss how these data can be helpful to advance our understanding of the topic under study?

-Is public health relevance addressed?

Reviewer #1: My criteria was included in the Instructions section.

Reviewer #2: It is an interesting work about a relatively rare disease. However, due to the small sample the study can only generate hypothesis. 

Considering that yellow fever is a vaccine-preventable disease and São Paulo is an area with a yellow fever vaccine recommendation, would be interesting to know about the vaccine status of the study subjects. Is this information available?

--------------------

**Editorial and Data Presentation Modifications?**

Use this section for editorial suggestions as well as relatively minor modifications of existing data that would enhance clarity. If the only modifications needed are minor and/or editorial, you may wish to recommend “Minor Revision” or “Accept”. 

Reviewer #1: None.

Reviewer #2: Please note that some terms need to be revised:

In the line 137, please review the term mortality (it’s probably lethality). 

In the line 141, please review the term mortality rate (maybe “case fatality rate”). 

Line 257 – lab results WERE. 

In the line, 394, please review the reference. “Prof. Alexandre Vranjac” is the name of the Centro de Vigilância Epidemiológica, not the author of the reference.

Data did not mention if the subjects were vaccinated – maybe it is an important information.

--------------------

**Summary and General Comments**

Use this section to provide overall comments, discuss strengths/weaknesses of the study, novelty, significance, general execution and scholarship. You may also include additional comments for the author, including concerns about dual publication, research ethics, or publication ethics. If requesting major revision, please articulate the new experiments that are needed.

Reviewer #1: The manuscript present a unique opportunity to evaluate the convalescent phase of a uncommon disease such a Yellow fever. As this disease presents sporadically, the chance to take direct measures of clinical parameters presents in a constrict time window that must be used, in order to better understand how the pathophysiology develops and have under-notice effects on this survivors.

With a reasonable number of UCI survivors of a disease with such a high reported mortality rate, the authors deliver a compelling argument substantiated with rigorous measurements and statistics.

All patients signed the informed consent and the projects was approved by the corresponding ethics committee.

The conclusions are interesting and solid.

Reviewer #2: (No Response)

--------------------

PLOS authors have the option to publish the peer review history of their article (what does this mean?). If published, this will include your full peer review and any attached files.

If you choose “no”, your identity will remain anonymous but your review may still be made public.

**Do you want your identity to be public for this peer review?** For information about this choice, including consent withdrawal, please see our Privacy Policy.

Reviewer #1: No

Reviewer #2: No

Figure Files:

While revising your submission, please upload your figure files to the Preflight Analysis and Conversion Engine (PACE) digital diagnostic tool, https://pacev2.apexcovantage.com. PACE helps ensure that figures meet PLOS requirements. To use PACE, you must first register as a user. Then, login and navigate to the UPLOAD tab, where you will find detailed instructions on how to use the tool. If you encounter any issues or have any questions when using PACE, please email us at figures@plos.org.

Data Requirements:

Please note that, as a condition of publication, PLOS' data policy requires that you make available all data used to draw the conclusions outlined in your manuscript. Data must be deposited in an appropriate repository, included within the body of the manuscript, or uploaded as supporting information. This includes all numerical values that were used to generate graphs, histograms etc.. For an example see here: http://www.plosbiology.org/article/info%3Adoi%2F10.1371%2Fjournal.pbio.1001908#s5.

Reproducibility:

To enhance the reproducibility of your results, we recommend that you deposit your laboratory protocols in protocols.io, where a protocol can be assigned its own identifier (DOI) such that it can be cited independently in the future. Additionally, PLOS ONE offers an option to publish peer-reviewed clinical study protocols. Read more information on sharing protocols at https://plos.org/protocols?utm_medium=editorial-email&utm_source=authorletters&utm_campaign=protocols

References

Please review your reference list to ensure that it is complete and correct. If you have cited papers that have been retracted, please include the rationale for doing so in the manuscript text, or remove these references and replace them with relevant current references. Any changes to the reference list should be mentioned in the rebuttal letter that accompanies your revised manuscript. If you need to cite a retracted article, indicate the article's retracted status in the References list and also include a citation and full reference for the retraction notice.

# Author comment: Author response to Decision Letter 0

**DOI:** 10.1371/journal.pntd.0009594.r002

10 Jun 2021

Attachment Submitted filename: Response Letter to the Editor and Reviewers.docx Click here for additional data file.

# Editor report: Decision Letter 1

**DOI:** 10.1371/journal.pntd.0009594.r003

**Authors:** Elizabeth J Carlton, Michael R Holbrook

24 Jun 2021

Dear Neves,

We are pleased to inform you that your manuscript 'Staging liver fibrosis after severe yellow fever with ultrasound elastography in Brazil: A six-month follow-up study' has been provisionally accepted for publication in PLOS Neglected Tropical Diseases.

Before your manuscript can be formally accepted you will need to complete some formatting changes, which you will receive in a follow up email. A member of our team will be in touch with a set of requests.

Please note that your manuscript will not be scheduled for publication until you have made the required changes, so a swift response is appreciated.

IMPORTANT: The editorial review process is now complete. PLOS will only permit corrections to spelling, formatting or significant scientific errors from this point onwards. Requests for major changes, or any which affect the scientific understanding of your work, will cause delays to the publication date of your manuscript.

Should you, your institution's press office or the journal office choose to press release your paper, you will automatically be opted out of early publication. We ask that you notify us now if you or your institution is planning to press release the article. All press must be co-ordinated with PLOS.

Thank you again for supporting Open Access publishing; we are looking forward to publishing your work in PLOS Neglected Tropical Diseases.

Best regards,

Michael R Holbrook, PhD

Associate Editor

PLOS Neglected Tropical Diseases

Elizabeth Carlton

Deputy Editor

PLOS Neglected Tropical Diseases

***********************************************************

# Editor report: Acceptance letter

**DOI:** 10.1371/journal.pntd.0009594.r004

**Authors:** Elizabeth J Carlton, Michael R Holbrook

15 Jul 2021

Dear Neves,

We are delighted to inform you that your manuscript, "Staging liver fibrosis after severe yellow fever with ultrasound elastography in Brazil: A six-month follow-up study," has been formally accepted for publication in PLOS Neglected Tropical Diseases.

We have now passed your article onto the PLOS Production Department who will complete the rest of the publication process. All authors will receive a confirmation email upon publication.

The corresponding author will soon be receiving a typeset proof for review, to ensure errors have not been introduced during production. Please review the PDF proof of your manuscript carefully, as this is the last chance to correct any scientific or type-setting errors. Please note that major changes, or those which affect the scientific understanding of the work, will likely cause delays to the publication date of your manuscript. Note: Proofs for Front Matter articles (Editorial, Viewpoint, Symposium, Review, etc...) are generated on a different schedule and may not be made available as quickly.

Soon after your final files are uploaded, the early version of your manuscript will be published online unless you opted out of this process. The date of the early version will be your article's publication date. The final article will be published to the same URL, and all versions of the paper will be accessible to readers.

Thank you again for supporting open-access publishing; we are looking forward to publishing your work in PLOS Neglected Tropical Diseases. 

Best regards,

Shaden Kamhawi

co-Editor-in-Chief

PLOS Neglected Tropical Diseases

Paul Brindley

co-Editor-in-Chief

PLOS Neglected Tropical Diseases
